# Researches on Operative Midwifery

**Published:** 1841-07-01

**Authors:** 


RESEARCHES ON OPERATIVE MIDWIFERY, &c., with Plates. By *Fleetwood Churchill*, M.D. Dublin, 1841, pp. 360.

THE present work of Dr. Churchill contains seven essays upon Operative Midwifery, one upon the Funis Umbilicalis, and a Report of the Dublin Western Lying-in Hospital: to these are appended a pretty copious midwifery bibliography, and plates of the various obstetrical instruments.

Midwifery, in its transition from an art to a science, has had to encounter many discouragements in this country. First, its very name, "man-midwifery," is a rude jargon in sound, and derogatory in its etymology. Then, in the place of assisting in elevating the character and position of its professors, a portion of the profession has by its influence and conduct contributed to a contrary result ; it is only at the present day, (among other evidences of a commencing, but tardy, appreciation of their true position and interest) that the College of Physicians has rescinded the prohibition of their Fellows to practise as accoucheurs. Add to all this, the utter want of tests as to proficiency on the part of those who undertake its responsible duties, which may, indeed, be legally assumed by any old wife or chemist's apprentice. Surely it is time to consider whether we shall continue to deliver up to any ignoramus, who may have the hardihood to undertake them, the treatment of some of the most fearful emergencies to which the fairest portion of the creation are liable. Still, in spite of all these obstacles, midwifery has flourished in this country; and we can, with equal pride, point to practical results, and to the list of distinguished men who have practised it.

Necessarily much of the first portion of this book is statistical, and we quite agree with Dr. Churchill in the following passage:—"I do not wish to overrate the value of statistics, at the utmost they only afford an approximation to the truth, owing to the numerous drawbacks from their exactness—but, nevertheless, I cannot doubt that they are of considerable value, and are, at least, available in pointing out the relative importance of each operation." Medical statistics have been as much overrated on the one hand, as they have been inefficiently appreciated on the other. Unquestionably, they are open to numerous fallacies, and the *cæteris paribus* is of difficult attainment. But if in any one branch of the profession they are likely to prove useful, it is in the obstetrical; as in it the whole facts of the case are more easily brought into view, and hence are susceptible of a more accurate comparison and generalization.

### I. Induction of Premature Labour.

This, one of the greatest improvements in modern midwifery, has been received in a very different manner in our own country and in France. Not rashly introduced by our predecessors among the allowable operations, but received after calm deliberation, it has since obtained the sanction and approval of almost every name of note among us. The reason is plain, the cavils and sophisms of divinity doctors entered not into the deliberations, but the dictates of common sense prevailed. Our neighbours, on the contrary, cramped by the mandates of the Sorbonne, and permitting themselves to become involved amid the mazes of priestly casuistry, have turned with horror from the idea of interrupting Nature's operations, and calmly proceeded to the performance of the alternative (with them)—the Cæsarian section.

The attention of practitioners was first drawn to this operation by observing accidental premature labour prove, in some cases, the means of preservation of the life of a child, which, owing to the degree of pelvic distortion present, could never have been born alive or unmutilated at the full period. By the artificial induction of this process then at a viable period of the child's existence, a chance would seem to offer itself for its preservation, while the mother would be saved the horrors and dangers of the operation of craniotomy. The operation, then, in its conception was most plausible, and in its results has proved most satisfactory. From among the great number of cases now referred to by Dr. Churchill, he finds that more than one half the children were saved, and thus, at all events, giving them the same chance of living possessed by seven months' children; while, again, in regard to the mother, her life is placed in much less peril than it is in those cases which, left to the full time, necessitate the operation for lessening the size of the fœtal head. Thus, out of 161 cases but 8 mothers died, and 5 of these from causes unconnected with parturition.

In reference to the *comparative utility* of the operation, Dr. Churchill has the following passage.

"It is peculiar to midwifery operations, that they form ascending series, increasing in gravity from the simplest to the most severe—no two being equal; and therefore, in considering the suitability or practicability of any one, we do so with the knowledge that, if the one we prefer does not succeed, we must have recourse to another more severe and more dangerous. An example will make my meaning clear. If, in any given case, we attempt to deliver with the forceps, but are not able to succeed, we must subsequently have recourse to the perforator; there is no other method, of *only equal* severity with the forceps, which we can try. Or again, if craniotomy and evisceration will not render the transit of the child possible, we have no resource but symphyseotomy or Cæsarean section.

Thus, the *alternative* of any operation in midwifery is not one of *less*, or even of *equal* danger, but necessarily one of a *more serious nature*, and consequently we cannot estimate the utility of any obstetric operation fairly, if we consider it by itself; a just appreciation involves a due estimate of its alternatives." 12.

Applying these remarks to the cases in question, he states, that by the use of the perforator about one in five of the mothers perish. By the Cæsarean section 1 in $2\frac{1}{3}$ mothers, and 1 in $3\frac{1}{2}$ children are lost, and by symphyseotomy one third of the mothers have been lost (those recovering even, often continuing great sufferers), and only one half the children were saved. "If then, to the *absolute* advantages of the operation proposed, be added the *comparative* gain from avoiding these terrible *alternative* operations, we may form a tolerably correct estimate of the *utility* of the induction of premature labor."

In considering the cases to which this operation is applicable, the ascertaining the relative sizes of the pelvis, and the fœtal head in its progressive development, is of importance. Dr. Churchill presents tables of these.

"It will at once be observed," he says, "that there are two measurements of the pelvis which limit the operation: if the pelvis exceed the greater measurement, the operation is uncalled for; and if less than the least, it will not succeed in saving the child. The smallest of these diameters appears to be about $2\frac{1}{2}$ inc. and the greater $3\frac{1}{4}$. If the pelvis, in its sacro-pubic diameter, be less than the former, a '*viable*' child will not pass, and it is generally admitted that a living child may be propelled through a pelvis whose antero-posterior diameter is $3\frac{1}{4}$ inches." 16.

Other cases, besides that of a deformed pelvis, may demand and justify the induction of premature labor, as exostosis, or fibrous tumors of the pelvis, as also when the uterus itself is the seat of fibrous tumors; which, during the latter months of pregnancy, take on, as described by Ashwell, a more active state of diseased action, often terminating in the death of the mother.

Of another class of cases, in which the saving the child's life is out of the question, Dr. Churchill observes;—

"But there are cases in which the distortion is so great as to render the passage of a seven months' child impossible, and others still worse, where no reduction of the child's bulk will enable it to pass. I do not see why abortion should not be induced at an early period in such cases. The life of the child must inevitably be sacrificed, and the safety of the mother alone regarded; and surely, after the calculations I have adduced, it cannot be pretended that Cæsarean section, the *alternative* in these cases, offers such a chance to the mother and child as would justify our preferring it." 20.

We agree with the author, that the objection offered to this suggestion, that it would be "opening a wide door to the abuse of this operation," is futile; the fear of the operation being perverted to improper cases or for improper purposes, must not deter us from having recourse to it in others, in which its use is clearly justifiable. Moreover, no medical man should, *under any circumstances whatever*, perform it without consulting a brother practitioner. The induction of premature labour has been recommended in various of the diseases of pregnancy, when these have arrived at the point of threatening the patient's life, such as vomiting, convulsions, hæmorrhages, &c.

Five different methods of effecting this operation have been recommended, viz.—1. Abdominal friction, warm baths, &c.; these are rarely of any utility. 2. The separation of the membranes for two or three inches around the os uteri; this plan, advocated by Hamilton and Conquest, is supposed by them and others to be safer for the infant, than when the membranes are punctured. 3. The puncturing the membranes is a more certain mode of proceeding than the former; it was employed by Dr. F. Ramsbotham in 36 cases, and of these 21 children were born alive. 4. The dilatation of the os uteri by means of a piece of sponge placed within it, and maintained there by a plug in the vagina. This plan, proposed by Brünninghausen and Klugè, is much approved of by Velpeau, although not deemed more certain by some authors than rupturing the membranes. 5. Ergot of rye has been of late extensively employed, but more children have been lost when it has been had recourse to, than when the other means have been employed.

Although instances are on record of children proving viable prior to the 7th month, yet that is the earliest period we must calculate upon in practice; and, to insure the attainment of this period, the author recommends we should, in estimating the probable age of the fœtus, "allow an additional fortnight, to rectify any error in the calculation of the period of pregnancy." A nurse with small nipples should be at hand to furnish nourishment to the child.

According to Dr. Merriman's statement, preternatural presentations occur as often as 17 times in 78 cases of premature labour: he suggests, that, supposing such should be detected, it would be better to wait a few days in the hopes of a spontaneous rectification taking place. But the author agrees with the objections of Hamilton and Dewees to this practice, grounded upon the great difficulty of ascertaining with certainty, at this early period of pregnancy, what part really presents, upon the greater ease with which the child is turned in the inverse proportion to its weight, and upon the great doubt which must exist as to the spontaneous alteration of the position of the child in a few days.

The next six essays, although they comprise the greater portion of the book, will not occupy us long. The author in these, has given a very good historical account of the various operations in midwifery, but has added little of personal or practical remarks that we can present to the reader.

### II. VERSION OR TURNING.

"Thus we see that the records of English practice yield 39,539 cases, and 147 cases of version—or about 1 in 269; French practice, 37,479 cases, and 400 cases of version—or about 1 in 93½; and German practice, 21,516 cases, and 337 cases of version—or 1 in 63⅘. The whole number of cases is 98,534, and of version, 884—or about 1 in 111½. It is not easy to make out a satisfactory table, shewing the dangers of the operation to the mother and child, from the want of details. Many writers do not mention whether any of the mothers died, and some omit the result as regards the child. I cannot forbear expressing my estimation of the minuteness and accuracy of Dr. Collins's statements, and the excellence of the tabular views he has given. In the following table (we have not space for it) I have taken all the numbers upon which I could depend, and though the list is not extensive, I believe that the average mortality will be found pretty correct. Thus, in 169 cases, 11 mothers died, or 1 in 15. I do not give this result as the exact mortality of the *operation*, because it is evident that the deaths in some cases may have been owing to the cause which demanded the operation—as in placenta prævia; but as we find that even in several of these cases, the fatal termination was evidently more owing to the operation than to the hæmorrhage, I am inclined to think the calculation not very far from the truth. However, any erroneous inference from these statistics, will be guarded against by the recollection of the various and serious accidents which require the operation.

In 542 cases, where the result to the child is detailed, 182 children were lost, or rather less than 1 in 3. To a certain extent the same observations apply to this calculation of the mortality amongst the infants, and similar allowance must be made. One in four is stated as the mortality in footling cases, which must evidently be below the proper estimate of version cases." 52.

The objects of version or turning, are either "to place the head in a more favourable relation to the pelvis, or to substitute the head for some other presentation" (cephalic version) : or, "to substitute the inferior extremities for some other less favourable presentation" (podalic version). As to *cephalic version,* much difference of opinion exists as to the eligibility and practicability of its performance. The rectification of malposition of the head by means of the fingers is well known to be often practicable at an early stage of labour; but the substitution of the head for other presenting parts is a matter of far greater difficulty. Velpeau, however, decribes it as very possible ; and Dr. Churchill says, that the cases in which it can be employed may be divided into two classes.

"First—where the pelvis is of sufficient size, and nothing but the *malposition* of the child's head calls for interference;—2nd, in certain *malpresentations,* such as the neck or shoulder, and perhaps in a few arm cases, if the uterus be not strongly contracted, and especially if the waters have not escaped. It is evidently not calculated for any case where prompt delivery is necessary." 56.

After describing the ordinary mode of rectifying the position of the head, the author thus continues.

"Wigand has stated that it is possible, before the waters have escaped, to change the position of the head, or even the presentation, by external abdominal manipulations. Velpeau confirms this from his own experience, and something similar is stated by Sennert and Martins. Riecke has also related several such cases. Dr. Burns in a note to his 9th edition, states, that 'Mr. Buchanan, of Hull, informs me that he succeeded in one instance lately, where the left side of the breast of the fœtus lay diagonally over the pelvis, with the head forward, in bringing the head right, by making the patient kneel and raise the breech, whilst the shoulders were brought as low as possible. The water had not been discharged. The situation of the head, when it came down, was made more favourable by the finger. The child was alive.'" 63.

In regard to the operation of turning, properly so called, the author contributes no additional information, his account being avowedly compiled from the various standard works on the subject. He makes no allusion whatever, to the interesting subject of the spontaneous evolution of the fœtus in transverse presentations.

### III. Instrumental Labour.

Two classes of cases may require the aid of instruments, namely, the one arising from a deficiency of room in the pelvic cavity, or the too great size of the fœtal head; and the other from defective uterine energy. The first class of cases, although presenting, frequently, difficulties in the decision of the course to be adopted regarding them, are more easily determined upon than the latter.

"But if we turn to the class of cases where the abnormal deviation from natural labour is caused by the inefficiency of the uterine efforts, we shall find the difficulty much greater. We have no standard for ascertaining the adequacy of the pains, nor for deciding upon the necessity of interference, except that which long experience gives to individuals. Besides, the question is not merely whether the unaided efforts of nature may not, after an indefinite length of time, accomplish the delivery; but whether the process will be completed before the constitution of the patient shall have suffered more than the ordinary shock. A patient may be delivered naturally, and yet die of the labour. In some cases there can be no doubt; the deficiency of uterine power, or the existence of constitutional disturbance is so marked, that prompt assistance of some kind is imperatively demanded. But in another class, these indications are less plainly marked; the pains cannot be said to be altogether inefficient; they might even complete the labour, if sufficient time could be allowed; but the patient is beginning to suffer, constitutionally or locally, from the prolongation of labour; and there are evidences that more danger may result from delay than from interference. Now, in these cases, where the power is inefficient, or altogether deficient, we have good ground for interference, provided we possess instruments which neither injure mother nor child, and which, from their mechanical arrangements, are calculated to assist feeble pains, or to supply their absence. For those cases where the deviation is dependent upon physical disproportion, it may be necessary that one life should be sacrificed to secure the other; and all that can be required is, that the instruments used be accurately adapted to secure the latter, as well as to effect the former.

Thus, whether we examine the cases we meet with, and decide upon the instruments required; or whether we classify the instruments we possess, we shall find them naturally divided into—1st. Those which are not intended to injure either mother or child, as the vectis and forceps; and 2nd. Those which are employed in the destruction and extraction of the child, but which are not intended to injure the mother, as the perforator and crotchet." 73.

### IV. The Lever or Vectis.

A meddlesome midwifery is a bad midwifery, says an eccentric but most able author, and the indiscriminate use of the lever by many practitioners justifies his remark. This instrument is most unfortunately named, for, as every lever must have its fulcrum, so do the pelvic bones present one, but too convenient for the hasty or ignorant operator. Our author thus describes the case he thinks best adapted for its employment.

"The case which appears to me most suitable for the use of this instrument, and in which the probability of success is greatest, is that which I have sketched at the commencement of this essay, when the head having descended into the pelvic cavity, is arrested in its progress, not by any mechanical impediment, but by the inefficiency (not absence) of labour pains, and when the patient is beginning to show symptoms of constitutional or local disturbance. This condition does not take place until the second stage of labour has lasted some time, and as, after these symptoms have shown themselves, there is danger to the patient in further delay, it is important to obtain aid...... Now as there is supposed to be space enough, and pains though feeble, a slight additional force will often succeed in bringing the infant into the world at once. As there is nothing in the nature of the operation to add to the danger (?), and especially as the tractile force will probably be sufficient, it seems peculiarly suitable to this case; and I may add, that all the testimony I can collect is in favour of its application." 97.

The lever may be used sometimes in rectifying the position of the head, and in expediting the labour in convulsions, &c.; but it is inadmissible in cases of impaction. As to whether preference is to be given to the lever or forceps, Dr. Churchill thus expresses himself:—

"Each writer has taken up this subject too much as a partizan. To compare their utility in certain cases, is little more than a waste of words; as, for example, where the pains have ceased, or where compression is required to extricate the head of the child. In such cases, the vectis is of no use, and it would be highly reprehensible to employ it. But, where there is room, and when the pains persist, there the vectis, being sufficiently powerful, has this signal advantage, that there is but one blade to be introduced, and but the thickness of that one blade added to the child's head. It is possible that the single blade may be able to act where the bulk of two would render extraction impossible. One point I must notice, which has been urged in favour of the vectis, viz., the *secresy* with which it may be used. Now this I consider a decided disadvantage." 99.

### V. The Forceps.

Almost every accoucheur of celebrity has thought it necessary to modify the form of this instrument: the author figures more than eighty of these varieties—a tolerable good proof of the difficulty even able hands have experienced in its employment. "For myself," he says, "I prefer the long or short forceps with the single curve, with the blades and fenestra somewhat narrow, and approximating, so as to allow of a firm degree of pressure; with their edges smoothly levelled off; and the blades sufficiently strong to prevent their springing, but not so thick as to add unnecessarily to their bulk. The hand that is to use the instrument is, however, of more importance than the instrument itself, of which it may be observed with truth 'that which is best administered is best.'"

As to the average frequency with which this operation is had recourse to, Dr. Churchill presents three tables, giving the following conclusions:—

Among British practitioners, 120 forceps cases occurred in 42,196 cases of labour—or about 1 in 351½. Among the French, 277 in 44,736 labours—or 1 in 162. And among the Germans, 1702 in 261,224 labours—or about 1 in 153½. In all, 2099 forceps cases in 348,156 cases of labour—or about 1 in 165. As to the *results* of the operation, only an approximation can be arrived at, since in some of the reports referred to, these have not been stated; and in others it has not been distinguished how far these may have arisen from other causes than the operation. Out of 294 forceps cases in British practice, 14 mothers, or 1 in 21, were lost. Among the French and Germans, in 479 cases, 35 mothers, or about 1 in 13½, were lost. Of children, about 1 in 4½ died in Britain, and 1 in 5 on the Continent.

In our author's observations upon the advantages attendant upon this instrument, the cases in which it is applicable, and the mode of its application, we find no novelty, nor anything to extract; the following caution should be written in letters of gold:—"I must also premise, that in no case is the forceps (or indeed any instrument) to be applied, until we are perfectly satisfied that the obstacle cannot be overcome by the natural powers, with safety to mother and child."

### VI. The Perforator and Crotchet.

The operation of craniotomy is of so revolting a character, that one cannot feel surprised at the prejudice against its performance which prevails in the minds of many. But it is the province of true humanity even to inflict a lesser evil for the prevention of a greater. The circumstances under which the operation is required and practicable are thus stated.

"The case presupposes, on the one hand, actual disproportion, sufficient to prohibit the passage of the fœtal head, even when compressed; and, on the other, that the distortion is not so great as to prevent the extraction of the child when mutilated. Dr. Osborn states that, when the bones approach much nearer to each other than three inches, it is utterly impossible for a living child at full maturity by any means to pass. He fixes upon 2¾ inches, as the diameter rendering craniotomy necessary. M. Alphonse Le Roi says that 3¼, Dr. Aitkin 3, Dr. Joseph Clarke 3½, Dr. Burns 3¼, Dr. Ritgen 2, and Dr. Busch 2½ to 3 inches, is the smallest antero-posterior diameter through which a living child can pass. As to the other limit of the operation, that is, the smallest diameter through which a child can be extracted after craniotomy, Baudelocque says that the crotchet is inadmissible when the diameter is only 1⅔ of an inch; Dr. Dewees when it is less than 2;—Dr. Hull and Dr. Burns believe that it may succeed when the diameter is 1¾; MM. Gardien and Hamilton when it is 1½; and Dr. Davis when it is 1 inch." (Dr. Osborn considers 1½ in. space enough, provided time be given for the softening of the child's body by putrefaction, and the cranial bones are picked out.) 169.

Dr. Churchill presents tables of the average frequency of this operation, whence it would appear to have been performed in Britain 181 times in 41,434 cases of labour—or about 1 in 228; in France, 30 times in 36,169 cases—or 1 in $1205\frac{2}{3}$; in Germany, 132 times in 256,655 cases—or 1 in $1,944\frac{1}{3}$. In estimating these numbers, the reader must bear in mind, that the axiom among British practitioners, that the child's life is to be unhesitatingly sacrificed when the mother's safety demands it, is not so implicitly received on the Continent, and hence the Cæsarian operation is often resorted to there, when the crotchet would be employed by ourselves.

The mortality amounted to 52 in 251 cases—or 1 in 5.

"At first sight one would expect the mortality among the mothers to be less, after the use of the crotchet than the forceps; but the result of these investigations shows the reverse to be the case. The only explanation I can give is founded upon the natural unwillingness of every humane practitioner to destroy life—the consequence of which feeling, is the delay of the operation so long as there is a hope of evading it. This delay, however, is unfavourable to the mother, and when at length the operation is performed, although it may have been less severe than delivery by the forceps, yet her condition rendered her much more susceptible of injury from it." 173.

The cases in which this operation is allowable, consist of those instances of distortion or diminution of the pelvic cavity, through which an entire child could not pass, when the head of the child is hydrocephalic, and under a variety of circumstances in which delivery is urgently demanded, and is not practicable by means of the forceps. We think Dr. Churchill should have dwelt a little upon the signs of the death of the child, for, although in most cases in which the operation is to be undertaken, this must become only a secondary consideration, yet it is at least a satisfaction when the death can be reasonably supposed to have occurred.

We would suggest to the author, in the event of his work reaching another edition, that it would be exceedingly useful to present a digest of the facts upon record regarding the use of the *ergot of rye* in tedious parturition. Not only is the subject interesting and important in itself, but it is especially so in relation to instrumental labour, as there can be no doubt of the great diminution in the number of lever and forceps cases, since the introduction of that drug into midwifery practice; but, many practitioners are of opinion, that the number of still-born children is much greater, than when tedious cases are left entirely to nature; our own experience, as far as it goes, confirms this opinion; but, what may be the proportion of children born alive when the ergot is used, and when instruments are employed, we have no idea of, nor is a just one likely to be obtained, until midwifery statistics are more copious and more exact; this they might easily be rendered if every practitioner would be at the pains of noting down the leading facts of the cases which come under his care, and from time to time communicating them to the profession.

### VII. THE CÆSARIAN SECTION.

This operation, never received with any favour in this country, has been performed very frequently on the Continent. Of course there are cases in which, from the narrowness of the pelvis, even a mutilated child cannot be transmitted, but these are rare, and in most instances ought to have been anticipated by the induction of premature labour. Dr. Churchill has collected references to a large number of cases, and thus expresses himself:—

"These tables yield the following results: 1. Among British practitioners, in 40 cases, 11 mothers recovered and 29 died, or nearly three-fourths. 2. Out of 37 cases where the result to the child is mentioned, 22 were saved and 15 lost, or 1 in 2½. 3. Among continental practitioners, out of 369* cases, 217 mothers recovered, and 152 died, or about 1 in 2⅓. 4. Out of 187 cases where the result to the child is given, 138 were saved and 49 lost, or nearly 1 in 4. 5. Taking the entire number, which amounts to 409, we find that 228 mothers were saved,† and 181 lost, or about 1 in 2¼; and that out of 224 children, 160 were saved, and 64 lost, or about 1 in 3½........ After a careful examination of the cases on record, I think we may fairly conclude, that as so many women have recovered from the operation, it does afford a chance to both mother and child, and that, therefore, we may be justified in having recourse to it; but that, as the danger is much greater than from any other operation, we should not be warranted in performing it, if there were a prospect of success by other means. This, then, constitutes the sole *advantage* of the operation, that in *cases where we cannot deliver the patient by any other means, and when, consequently, both mother and child would inevitably die if left unaided, we may afford each a chance, by performing the Cæsarian section.*" 225.

### VIII. Symphyseotomy.

This operation originating in an erroneous idea that the symphisis pubis separates and thus enlarges the cavity of the pelvis, in difficult labour, after the sacrifice of several victims to hasty conclusions, has sank into that oblivion, which a due knowledge of the anatomy and physiology of the parts, might at once have consigned it to.

### IX. The Funis Umbilicalis.

This is a very interesting chapter.

*Growth, Length, &c. of the Funis.*—Although Hunter and Burns mention the 6th week as the time within which the cord is discovered, yet, Velpeau and Breschet state, that if the ovum be perfect, a cord is discernible from the earliest period, and our author coincides with these latter authorities.

---

* In 28 of these the women underwent the operation more than once.

† "I do not mean that so many mothers were saved from death by the operation, but that they were saved from the effects of the operation. No doubt many were really saved from death, which could not have been otherwise avoided; but we have proof that many could have been delivered by other means, inasmuch as they afterwards bore children naturally."

"It is generally visible about the 15th day, when it rather exceeds the length of the fœtus. Up to the 3d week it is thin and cylindrical; from the 3rd to the 9th it encreases in size, and we find two or three vesicular swellings with narrow intervals; the last swelling persisting the longest. It contains at this period the vitelline duct, with the omphalo-mesenteric vessels, a portion of the urachus, the fœtal intestines at its umbilical extremity, the blood-vessels, and some gelatine. About the end of the 2nd month the intestines are enclosed in the abdomen, and the vitelline duct and urachus are obliterated, so as scarcely to be detected afterwards." 262.

At the termination of pregnancy, the point of *origin* in the placenta is various. Osiander found out of 36 cases, in one only did the funis arise exactly from the *centre*, usually arising about two inches from the edge. This is contrary to our own observations made upon a considerable number of cases. The *length* of the cord is very variable; all lengths have been met with, from 2 inches, (Davis,) to 5 feet (Gardien). The author gives a table of 500 measurements which he has made in private and hospital practice. The conclusion from it is as follows.

"Thus we find that the most frequent length was 18 inches, the next 24, and the next to that 20 inches; so that we may agree with those authors who state the average length of the cord to be between 18 and 24 inches. Extremely short cords must be very rare, and scarcely to be calculated upon in practice—since out of 500 cases none were under 12 inches (6 were 12 inches). Only four were about 3 feet. I have no data by which to test the observation of Osiander, that male children have generally longer cords than females." 265.

After describing the structure of the cord, and detailing the evidence of its possession of nerves and lymphatics, the author alludes to various deviations from the normal conditions. When speaking of cases in which the funis and umbilicus are said to have been absent, he mentions the following deviation.

"A case of an acephalous fœtus recently occurred at the Western Lying-in Hospital, which had formed adhesions by the back of the neck to the placenta, from which the funis arose, and passing round the right side of the neck, was inserted into the depression between the face and neck, just about the spot where the angle of the jaw should have been, had there been no malformation. The vessels of the cord passed behind the clavicle and ribs, down into the chest and abdomen, and were there lost. There was a depression, or *cul de sac*, about the proper situation of the umbilicus. This is the most remarkable deviation from the usual course of the funis I have ever seen. Chaussier relates an instance where the placenta was attached to the liver, and another is on record in which it adhered to the abdomen." 273.

*Knots of the Cord.*—These are not unfrequently found, and may be single or double. Burns supposes that they may sometimes be formed during labour, but those to which allusion is now especially made, are probably formed during an early period of utero-gestation. Some authors consider them as very dangerous, or even fatal, but a contrary opinion held by others, is believed by Dr. Churchill to be the just one. As the cord has been sometimes knotted after death, in order to conceal infanticide, by presenting an apparent cause of death, it may become an important point to distinguish knots of the cord thus formed from those formed in utero.

"If the knot be moderately loose, and the vessels on either side varicose: if the injection pass easily, and the cord curls when untied, and the inside of the circle be flattened, we may conclude that the knot is of old standing, and also that it could not have caused the death of the fœtus. If, on the other hand, the knot be drawn very tight, so that the injection cannot pass; if there be no varicose state of the vessels; no flattening of the inner surface of the knot; and no curling round when untied, the evidence is in favor of the recent formation of the knot, either during the delivery, or subsequently, and more or less against the supposition of its having caused death." 278.

*Coiling of the Funis round the Child's Neck.*—"The funis may be coiled round the child's neck, extremities, or body, in consequence of its excessive length, (the author never met with it in funes of less than 18 inches, usually 24,) and the movements of the fœtus in utero. If the length of the cord be very great, several coils may be formed. I have quoted from M. Heritier, a case where it was five times round the neck. An artificial coil may be formed during the expulsion of the child, or in turning. As to the frequency of this occurrence, Richter of Moscow met 27 examples out of 624 cases; Siebold, at the Berlin Clinique, 21 out of 137 cases; and Kluge 63 out of 268. Dr. Ashwell mentions 30 cases of coiling in 649 cases. I give these on the authority of M. Velpeau, as I have not the documents to refer to. Out of 242 cases, of which notes were taken under my own superintendence, it occurred 63 times. Thus, in 1920 cases, there were 204 examples of coiling, or rather more than 1 in 9." 276.

*Position of the Umbilicus.*—This has been observed with minuteness in reference to legal medicine. The insertion of the funis, which is near the pubis at the early period of fœtal life, is said to be, by Chaussier, Montgomery, and others, in the centre of the body at birth in a full-timed fœtus. M. Moreau having examined 500 children at the Maternité in reference to this point, has, however, found the insertion only central in 4 cases. On an average it was found to be 8 or 10 lines below this point, while in a few instances of premature children the cord was inserted in the centre.

The cord has been supposed frequently to exert a considerable influence on *parturition.* Thus labour has been said to have been impeded by a too *short* cord. This has been supposed to be a cause of retraction of the head, of reversion of the uterus, and of rupture of the cord. The author thinks that the statistics of midwifery amply prove the truth of Naegele's remark, "that the cord is rarely, if ever, so short as to hinder labour, or to entail any serious consequences." So, too, the shortening of the funis produced by its coiling around the infant has probably little or no influence upon the labour. The funis may be *prolapsed* either at the beginning or during labour. In 92,017 deliveries this occurred 333 times, or 1 in every $276\frac{1}{3}$. Many circumstances have been supposed influential in producing this occurrence; such as a small child and abundant liquor amnii, a long funis, and according to Naegele, irregular shape, or irregular action of the uterus. "I may add, from my own observation, that I have found, in several cases of prolapse, that the *placenta* was situated *low down,* near the cervix uteri, and in some few others, that the *funis was inserted into the lower edge* of the placenta." In the *treatment* of this deviation, no general rule prevails; some practitioners endeavour, but with indifferent success, to return the cord, and retain it free from pressure by some mechanical contrivance; others have thought it better to expedite the labour by means of turning, or the forceps. "No matter what means are adopted, the mortality, especially in hospital practice,* will be very considerable: thus, out of 249 of the cases I have referred to, 155 were lost and 94 saved.

*Ligature after Birth.*—It would be thought a great omission in this country to neglect the due application of the ligature, and, where this has been done carelessly, fatal hæmorrhage has often resulted. Nevertheless, several physicians object to it as unnecessary, and as likely to cause congestion. The author has seen infants, in M. Capuron's wards, in whom no ligatures were applied. Doubtless, in a state of nature, when the cord is probably torn rather than cut, fatal hæmorrhage would be rare, as it is in those cases of ruptured funis which are sometimes met with in sudden deliveries; but when the cord has been cut, there must always be great risk of subsequent hæmorrhage, although this may not be manifest at the time of the incision. As to the second ligature, the author says—

"It is customary in these countries to tie the cord a second time nearer the placenta, with a view to prevent hæmorrhage, in case there should be a second child, and a vascular communication between the placenta. It is a matter of precaution, not of necessity. It is said by some French authors, that allowing the cord to bleed, facilitates the expulsion of the placenta. I am not prepared to speak decidedly upon this point, but I know that it weakens the cord, and unfits it for much traction." 292.

*Decadence of the Cord.*†—Of the period when this occurs Dr. Churchill has kept an account in 200 cases.

"In 1 case it fell on the 2nd day—in 4 on the 3d—in 20 on the 4th—in 52 on the 5th—in 81 on the 6th—in 24 on the 7th—in 10 on the 8th—in 7 on the 9th—in 1 on the 10th. Thus the 5th and 6th days are the ordinary periods of its detachment. My friend, Dr. Montgomery, informs me he has had one persistent until the 15th day, and Dr. Breen has seen one at the 13th day." 294.

### X. REPORT OF THE WESTERN LYING-IN HOSPITAL.

This is a statistical account of the cases which have occurred in this Institution during five years: such reports are very useful, and it is to be regretted that any individual, having it in his power to produce similar ones, should neglect doing so. A brief abstract may prove interesting.

During the five years 1705 females were delivered; 65 of these were abortions, and 1640 children at the full time. The number of children amounted to 1667, including 25 cases of twins and one of triplets: 123 were still-born or died soon after birth, of whom 18 were premature and 6 putrid. In 1525 cases the presentation was as follows:—in 1435 the head presented; 16 head and hand (1 died); 35 breech (14 died); 22 foot

* Many cases do not seek admission into the hospitals until a chance of safe delivery is diminished, or even until the cord has ceased to pulsate. Twenty-two such cases occurred out of the 73 unfavourable cases recorded by Collins.

† For an account of Billard's interesting observations upon this subject see Medico-Chir. Rev. Vol. 32. p. 382, April 1840.

(10 died); 9 arm (5 died); 7 funis (5 died); 1 placenta (died). *Flooding* occurred *before* labour in 4 cases;—3 were cases of accidental, and one of unavoidable hæmorrhage. Flooding occurred *after* labour in 21 cases; no patient being lost from this cause. *Convulsions* occurred in 3 cases; 2 recovered:—13 patients were attacked by *puerperal fever;* of whom 4 died. *Turning* was required in 11 cases, or 1 in 149: three of the children and all the mothers were saved. The *forceps* was employed in 3 cases, or 1 in 547; one child was putrid, the others were saved, as were all the mothers. *Craniotomy* was performed in 12 cases (1 in 136); one woman died, but she was not seen until her case was hopeless. Of the entire number (1705), 10 women died, or 1 in 170—of these, 1 died from prolonged second stage (owing to neglect) giving rise to fever; 1 from pulmonary disease, combined with the shock of an operation; 2 from mismanagement of attendants after delivery; 4 from puerperal fever; 1 from apoplectic convulsions; 1 from cause unrecorded.

This volume, like its predecessors, presents an interesting resumé of what is known on the subjects upon which it treats; and, with even less pretensions to originality than the former volumes, exhibits the meaning of the various authors quoted faithfully, and often in the very words employed by them. The chronological list of British and Continental works of Midwifery will be found useful to the student; but we think the Plates of the various instruments employed in obstetrical surgery, many of which are now obsolete, might have been omitted, tending, as they do, to encrease the price of the book without corresponding advantage. We strongly recommend the work to our professional brethren.

